# Plant-Growth-Promoting Bacteria

**DOI:** 10.3390/plants13101323

**Published:** 2024-05-11

**Authors:** Carmen Bianco

**Affiliations:** Institute of Biosciences and BioResources, National Research Council, 80131 Naples, Italy; carmen.bianco@ibbr.cnr.it

## 1. Introduction

Global food-production levels may soon be insufficient for feeding the population, and changing climatic conditions could further limit agri-food production [1,2,3,4]; it is therefore essential that agricultural productivity increases significantly in the coming decades. Agricultural production is moving towards adopting more sustainable and environmentally friendly approaches, one of which is the growing use of bacteria that promote plant growth, called plant-growth-promoting bacteria (PGPB) [5,6]. PGPB can help plants to cope with abiotic and biotic stresses and to persist even in the case of nutrient deficiency. The use of PGPB is also an important strategy for soil-pollution remediation, which leads to the enhancement of soil quality [7,8,9].

**Figure 1:**
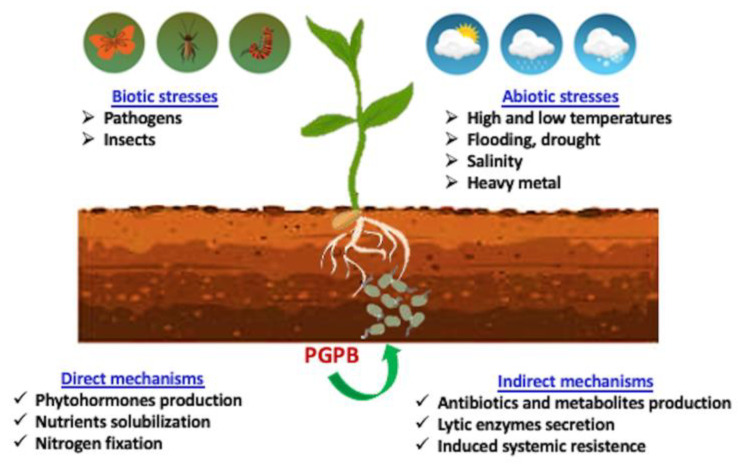
Schematic overview of the main mechanisms used by PGPB to stimulate plant growth.

The (direct and indirect) beneficial mechanisms of PGPB include the production of phytohormones, nitrogen fixation, phosphorous solubilization, and the sequestration of iron by bacterial siderophores. Indirect mechanisms refer to the bacterial traits that inhibit the functioning of one or more plant pathogenic organisms, including both fungi and bacteria (Figure 1) [10,11]. However, a comprehensive understanding of the mechanisms by which PGPB confer plant-adaptation benefits against various environmental stresses is still missing.

This Special Issue contains 12 research articles that focus on a wide range of plant species, such as model plants, crops, medicinal plants, vegetables, and trees, and discuss some of the mechanisms involved in PGPB. This Special Issue also contains four reviews that contribute towards a better understanding of the effects of PGPB on the response of host plants to biotic stresses and on the availability of essential nutrients like phosphate and iron. The authors contributing to this Special Issue have affiliations with institutions from several countries, including Belgium, China, Egypt, India, Italy, Norway, Portugal, Saudi Arabia, Russia, Spain, Thailand, and Ukraine.

Beyond the scientific significance of this Special Issue, I would like to highlight that some manuscripts, both among the articles and the reviews, have been produced by scientists from both Russia and Ukraine, thus demonstrating how scientific research can provide opportunities that allow for dialogue between nations at war. Otherwise, the objective of this paper is to describe the manuscripts published within this Special Issue, highlighting the main findings obtained from various studies; a brief description of each manuscript is provided below.

## 2. An Overview of this Special Issue’s Articles

Devarajan et al. [12], Redondo-Gómez et al. [13], and Bertani et al. [14] assess the potential of PGPB to promote rice growth in the context of climate-change. Devarajan et al. using the spray method for plant inoculation studied the effects of *Bacillus* strains on the drought tolerance of a susceptible rice cultivar, and examined the resultant changes in plant-stress-related physiological and biochemical parameters and stress-responsive genes. They found that, under drought-stress conditions, rice plants inoculated with the strain *B. megaterium* PB50 showed increased contents of total sugars, proteins, proline, phenolics, potassium, calcium, abscisic acid, and indole acetic acid. Greater panicle lengths, panicle weights, and 100-grain weights than that measured for irrigated control plants were found for rice plants foliar-sprayed with PB50. Redondo-Gómez et al. [13] tested the effects of five biofertilizers from halophytes on rice growth and physiological response under salinity stress and increased atmospheric CO_2_ concentration and air temperature. Showing that biofertilizers containing microbial consortia from halophytes improve rice growth and physiological response, the authors also showed that certain combinations of strains in a consortium might be effective in mitigating the negative effects of salinity and high CO_2_ concentration and temperature on rice plants. Following this, Bertani et al. [14] reported that the strain *Pseudomonas chlororaphis* ST9 was an efficient rice-root colonizer that becomes integrated into the plant’s resident microbiota, affecting the expression of several plant genes. They found that the rice gene encoding for metallothionein, a metal-binding protein involved in metal homeostasis, was always downregulated in inoculated plants at each time point, which was in agreement with previous data, suggesting that *P. chlororaphis* ST9 can be maintained at low stress levels.

In another paper, Alhammad et al. [15] investigate the impact of water management involving *Azospirillum brasilense* and *Rhizobium pisi inoculation* on wheat productivity and soil properties in dry regions. This study was designed to test the hypothesis that combining PGPR with a suitable irrigation method can improve plant growth and soil properties under limited water conditions. In their study, three water-management techniques were compared, using normal irrigation as a control as well as deficit irrigation and partial-root-drying irrigation. *A. brasilense* and *R. pisi* co-inoculation combined with partial-root-drying irrigation resulted in a higher number of grains per spike, a higher grain yield, and a higher crop-growth rate compared to rhizobacteria inoculation combined with the deficit irrigation. This increase was due to the higher production of growth hormones and higher leaf-area index under water-limited conditions. The greater leaf-area index also led to a higher chlorophyll content and a higher yield.

Cruz et al. [16] isolated and characterized bacterial strains from Sal Island (Cape Verde) and evaluated their osmotic tolerance and a number of their growth-promotion traits. The osmo-tolerant strains were used to inoculate maize plants, and the drought-tolerance of inoculated plants was evaluated by measuring their germination, growth, and physiological and biochemical parameters. The results presented in this study highlight the potential of osmo-tolerant bacteria to further increase the level of drought-tolerance in maize varieties, decreasing their dependence on irrigation, opening new perspectives to maize growth in drought-affected areas.

Three manuscripts in the Special Issue use *Arabidospis thaliana* as a model system to study the mechanisms of PGPB–plant interaction. Rodrigues-dos Santos et al. [17] utilized massive-sequencing techniques and biochemical assays to understand the plant’s metabolic regulation, underlining the interaction with this beneficial strain.

By using the model strain *Priestia megaterium* YCR-R4 and the model plant *Arabidopsis thaliana* Col-0 to carry out transcriptomic analysis, the authors found that many central metabolism-related genes were upregulated during plant–bacterium interactions. Using biochemical tests, they showed that the regulation of this relationship led to an increase in the total cellulose and lipids contents.

The results reported by Langill et al. [18] demonstrated that the selection of the core seed endophytic microbiome is crucial for host-plant fitness, highlighting the precarious symbiotic relationship it has with both the plant and seed. They hypothesized that introducing seed endophytes from *N. caerulescens* to the seeds of *A. thaliana* would improve the germination and overall plant health and biomass when *A. thaliana* is forced to germinate in marginal conditions. They also suggested that seed endophytes, being conserved generationally, are very important to plant health and germination success.

Averkina et al. [19] reported the use of three well-studied and characterized model PGPR strains, such as the wild-type and auxin-deficient strains *Azospirillum brasilense* Sp245 and FAJ0009, respectively, and the strain *Pseudomonas simiae* (WCS417r), to study the involvement of the protein Phosphatase 2A (PP2A) in PGPR–plant interactions. In their study, an *Arabidopsis* wild type and PP2A mutants were inoculated with the selected PGPR strains, and the resulting phenotype was analyzed. By comparing the results obtained for the different mutants, they suggested that the PP2A catalytic subunits C2 and C5 play a significant role in the response of the host plant to the bacterial inoculation.

Wiriya et al. [20] focused their study on the rhizosphere microbial community of oil crops. They investigated the abundance and diversity of viable bacteria and AM fungi dwelling in physic nut and sacha inchi rhizospheres; they state that soil fertility and physicochemical properties are the critical factors that influence the bacterial abundance and the community structure of AM fungi in both these rhizospheres. In pot experiments, when physic nut and sacha inchi were inoculated with either PGPR or AM fungi derived from both oil crops, they observed enhanced development and growth of these plants in most treatments, compared to the non-inoculated controls.

In the study of Li et al. [21], seedlings of the medicinal plant *P. polyphylla* var. *yunnanensis* were inoculated with various combinations of three dominant organophosphate-degrading bacteria (OPDB) (*Bacillus mycoides*, *B. wiedmannii*, and *B. proteolyticus)* isolated from the rhizospheric soil of *P. polyphylla* var. *yunnanensis*. Their effects on the yield and quality of the host plants were measured through the biomass of the rhizomes, the concentration of medicinal compounds, and the changes in the P forms in the soil. The authors found that the inoculation with consortia containing all the three OPDB resulted in the highest content of biomass, steroidal saponins, and Olsen-P.

A study conducted by Omar et al. [22] explores the potential plant-growth promotion of four *Streptomyces* strains and their role in enhancing cucumber growth and yield under greenhouse conditions. The plant-growth-promoting features, such as indole acetic acid (IAA) production, siderophore excretion, and solubilizing phosphate, of four *Streptomyces* strains (*Streptomyces* sp. strain HM2, *Streptomyces thinghirensis* strain HM3, *Streptomyces* sp. strain HM8, and *Streptomyces tricolor* strain HM10) from the Qassim region, Saudi Arabia, were evaluated in vitro. These strains were used to inoculate cucumber plants under greenhouse conditions, and their plant-growth-promoting effects on cucumber plants were then evaluated. Strain HM3, followed by strain HM8 and strain HM10, revealed the best results for plant height, the number of leaves per plant, root length, the number of fruits per plant, fruit length, fruit diameter, fruit fresh weight per plant, soil P after 21 DAT, and soil P at the end of the experiment.

Lastly, the study carried out by Bilous et al. [23] finds its context in the conservation and restoration of forest. The authors isolated ten endophytic bacteria from the tissues of unripe acorns of *Quercus robur* L, four of which were identified as *Bacillus amyloliquefaciens*, *Bacillus subtilis*, *Delftia acidovorans*, and *Lelliottia amnigena*. Analyzing the activity of the pectolytic enzymes causing the maceration of plant tissues, they found that the isolates, conditionally labelled as Q2 (*Bacillus subtilis*) and Q7 (*Bacillus amyloliquefaciens*), did not have the ability to macerate. The effect of potential growth-stimulating bacteria on *Q. robur* seedlings was studied by inoculation into the leaf tissue. The effect of the selected endophytic bacteria was compared with that of phytopathogenic and epiphytic bacteria, which cause injury to the surface of the leaves. After inoculation with isolates Q2 and Q7, and their complex (1:1), there were no signs of damage to the leaf plates in the inoculation zones. This was possible given the action of bioactive compounds, particularly phytohormones, that were synthesized by these endophytic bacteria species.

The four reviews featured in this Special Issue together provide an overview on siderophore-producing PGPB, phosphate-solubilizing bacteria (PSB), and endophytes as biocontrol agents.

The first review by Timofeeva et al. [24] concerns the siderophore-producing PGPB and the classification of different types of bacteria-produced siderophores that are promising for agriculture, and describes the life cycle of siderophores from their biosynthesis in the bacterial cell to the release of Fe from the Fe–siderophore complex into the plant. It also examines different methods of detecting siderophores and siderophore-producing bacteria. The authors highlight that many studies on the effect of siderophore-producing PGPB on plant growth and development revealed siderophore activity only via the chromium azurolsulfonate (CAS) test, which allowed them to establish the presence or absence of siderophore-producing activity. However, the analyzing the mechanisms of siderophore synthesis and their effect on plant growth and development is essential for the development of new strategies for rational farming. This review reports that, to date, neither the relationship between siderophores and their effect on plant growth and development nor the exact mechanism by which plants assimilate Fe with the help of bacterial siderophores are yet clear. The topics discussed in this paper demonstrate that further studies need to clarify the mechanisms of siderophore biosynthesis and to evaluate their physiological role. The authors also pointed out that a better understanding of this relationship could be useful for the development of new biofertilizers based on siderophore-producing bacteria.

The second review by Timofeeva et al. [25] discusses the diversity of phosphate-solubilizing soil microorganisms and the key mechanisms of phosphate solubilization, highlighting that the secretion of low molecular weight organic acids, which leads to a decrease in pH, is the main mechanism of microbial phosphate solubilization. The efficiency of solubilization depends on the strength and nature of the acids. Among them, solubilizing phosphates, gluconic acid, and 2-ketogluconic acid are the most common solubilizing agents for mineral phosphates. The process of phosphate mineralization also involves the activity of several groups of enzymes secreted by phosphate-solubilizing microorganisms. The enzymes that dephosphorylate phosphorester or phosphoanhydride bonds in organic compounds are non-specific acid phosphatases (NSAP), the most studied of which are phosphatases, which can be acidic or alkaline. The pH of the soils demonstrating phosphatase activity was acidic to neutral, indicating that acid phosphatases play a major role in this process. The potential for phosphate-solubilizing microorganisms to be used as biofertilizers to increase phosphorus bioavailability for the plant, promote sustainable agriculture, improve soil fertility, and raise crop yields is also discussed in this review.

In another review, Oukala et al. [26] summarize the diversity of bacterial endophytes and discuss the mechanisms through which they colonize the inner tissues of host plants. Another point of focus here is the ability of endophytes to enhance plant defenses. Endophytes may adopt several strategies to attenuate the negative impacts of pathogens and pests on their host. Those activities may be achieved by the direct inhibition of pathogens, since they share similar colonizing patterns and come into close contact with plants. The direct inhibition of pathogens is mainly mediated by the inhibitory activity of siderophores, antibiotics, cell-wall-degrading enzymes, volatile organic compounds, alkaloids, steroids, quinines, terpenoids, phenols, and flavonoids, or by the quenching signals of pathogens.

Otherwise, the indirect activation of plant defense associated with endophytes is performed by defense stimulation through induced systemic resistance (ISR) or endophyte-induced resistance (E-IR). Each interaction or group of interactions can develop different strategies inducing resistance, depending on the pathosystem. In general, the priming process depends on different hormonal pathways, such as the ethylene (ET) pathway. ET is considered a gaseous hormone that may influence physiological responses to the environment and stress. Bacterial endophytes could help to indirectly enhance the resistance of plants to stress by decreasing ET levels, especially during stress, when ET concentration increases.

Finally, the review by Kashyap et al. [27] summarizes strategies for the isolation and identification of endophytes, and covers different in vitro and in vivo screening methods to test them as biocontrol agents. In this review, the biocontrol mechanisms of endophytes and their potential application in plant-disease management have also been discussed; it emphasizes that registration and authorization are required before endophytes can be marketed and used as microbial biocontrol agents. Another point highlighted in this review is that, despite the multiple benefits of endophytes in plant-disease management, their uses in field conditions are limited. More studies on endophytes are required for screening stress-tolerant candidates for their translation from the lab to land.

## 3. Conclusions

The present Special Issue provides important practical insights into the different mechanisms that PGPB adopt to promote plant growth. The articles associated with this Special Issue highlight the crucial role played by PGPBs in protecting plants from biotic and abiotic stresses, which, due to increasing environmental challenges resulting from climate change, have significant effects on agricultural production. As Guest Editor, I am grateful to all the authors for choosing this Special Issue in which to publish their research. I hope that the findings reported in these papers will be useful for the development of research into plant-growth-promoting bacteria.
